# Rejuvenation in Deep Thermal Cycling of a Generic Model Glass: A Study of Per-Particle Energy Distribution

**DOI:** 10.3390/ma15030829

**Published:** 2022-01-22

**Authors:** Marian Bruns, Fathollah Varnik

**Affiliations:** Interdisciplinary Centre for Advanced Materials Simulation (ICAMS), Ruhr-University Bochum, 44801 Bochum, Germany; marian.bruns@rub.de

**Keywords:** atomistic simulation, aging, structural relaxation, rejuvenation, thermal cycling

## Abstract

We investigate the effect of low temperature (cryogenic) thermal cycling on a generic model glass and observe signature of rejuvenation in terms of per-particle potential energy distributions. Most importantly, these distributions become broader and its average values successively increase when applying consecutive thermal cycles. We show that linear dimension plays a key role for these effects to become visible, since we do only observe a weak effect for a cubic system of roughly one hundred particle diameter but observe strong changes for a rule-type geometry with the longest length being two thousand particle diameters. A consistent interpretation of this new finding is provided in terms of a competition between relaxation processes, which are inherent to glassy systems, and excitation due to thermal treatment. In line with our previous report (Bruns et al., PRR 3, 013234 (2021)), it is shown that, depending on the parameters of thermal cycling, rejuvenation can be either too weak to be detected or strong enough for a clear observation.

## 1. Introduction

When cooled below their glass transition temperature $T_{g}$, metallic glasses (MGs), alike other amorphous solids, undergo structural relaxation approaching an unreachable equilibrium state [1]. This process of releasing residual stresses via non-affine atomic rearrangements, i.e., *physical aging* is characterized by, e.g., a narrowing of distributed quantities such as the per-particle potential energy and a shift of their mean closer to the respective equilibrium value, in the case of potential energies toward lower values [2]. The well observed homogenization of the atomic structure during aging [3] leads to smaller variations of per-atom potential energies, resulting in a narrowing of their distributions. Local structural heterogeneities are known to be the carriers of amorphous plasticity [4,5]. A more relaxed (i.e., homogeneous) atomic structure is creating a more brittle fracture mode, shown by an increasing elastic modulus, a growing yield stress, and a lack of plasticity and ductility [6,7,8,9,10,11]. This hampers the application of glasses as structural materials as well as their stability during forming processes.

To improve the plastic deformability of metallic glasses, it has been proposed that mechanical [12] and thermal treatments [13,14,15,16] can be utilized to reintroduce stresses into the material, bringing it to a higher energy state and lowering brittleness This “reversing” of the effect of aging is called “rejuvenation”. Such effect is considered to be mediated by spatial heterogeneity in the systems’ response toward external manipulation of its state. In the case of thermal treatments, this is reflected in a spatial variation of the thermal expansion coefficient [17].

However, to date, the literature on effects of deep thermal cycling is rather controversial. While rejuvenation was reported in findings from experiments on Cu${}_{46}$Zr${}_{46}$Al${}_{7}$Gd${}_{1}$ and La${}_{55}$Ni${}_{10}$Al${}_{35}$ metallic glasses [13,18], other experimental studies showed that, depending on the material and its composition, both rejuvenation and aging [19,20] can occur.

The situation is not much clearer on the side of molecular dynamics (MD) studies. For a binary Lennard-Jones (LJ) glass, it is reported that thermal cycling is sensitive to the annealing state of the system, where rapidly quenched samples undergo relaxation upon thermal cycling but “well-annealed” ones show both aging for low and rejuvenation for high temperature intervals (cycling amplitudes) [21]. Experiments on a Zr-based metallic glass, on the other hand, report that “over-aged” samples do not show any sign of rejuvenation [18]. Molecular dynamics simulations of these latter systems show a strong dependence on the glass’s preparation history and details of the deep-temperature cycling procedure [22]. Although rejuvenation was observed in these simulations, it was argued that the system size is too small for the proposed heterogeneity of thermal expansion coefficient [17] to play a significant role here.

Indeed, based on molecular dynamics studies of volumetric strain and the related stress distribution in a Cu${}_{50}$Zr${}_{50}$ model glass, it has been suggested that the particle number in this system must exceed a threshold of roughly five thousands for heterogeneity in thermal expansion to become an effective yielding mechanism and give rise to rejuvenation [23].

We have recently investigated the effects of thermal cycling on a simple model system, the Kob–Andersen binary LJ glass but could not observe any detectable sign of rejuvenation in none of the cubic simulation boxes containing sixteen thousands up to 1.2 million particles [20]. However, while calorimetry experiments are in line with this absence of detectable effects of deep thermal cycling [20], recent tracer diffusion experiments performed on Pd${}_{40}$Ni${}_{40}$P${}_{20}$ bulk metallic glass samples seem to indicate enhanced diffusion upon thermal cycling [24].

Here, we revisit this issue with the same generic glass model as investigated in [20] but tune two important parameters. On the one hand, we increase the aging time prior to cycling (thus producing a more relaxed initial state) and at the same time enhance the cycling frequency. Thereby, we enlarge the separation of time scales between aging and thermal cycling. On the other hand, we increase the linear dimension of the simulation box by roughly a factor of fifteen to better explore effects arising from structural heterogeneity. Since a cubic geometry makes such an undertaking computationally prohibitively expensive, we have resorted to a slab-shaped geometry. This choice is encouraged by our previous study, where the use of a long linear dimension was found to be instrumental in unrevealing the spatially slowly varying structural heterogeneity, which occurs on the scale of many hundreds particle diameters [25].

This approach proves effective in revealing strong rejuvenation effects upon deep-temperature cycling regarding per-particle energy distribution. Through comparisons of the slab-type geometry with a cubic system containing the same number of particles, we show that the linear dimension plays a key role here, as a much weaker effect is found in the cubic case, which has a significantly smaller linear dimension for the same number of particles.

In the next section, we introduce the simulated model and describe important details of the deep-cooling procedure. Simulation results are then presented in Section 3. A conclusion and outlook compiles our most important findings and closes this manuscript.

## 2. Computational Details

### 2.1. Model

For the glass former, we use the well-known Kob–Andersen (80:20) binary Lennard-Jones mixture [26,27]. In this model, pairs of particles of types α and β interact via a binary LJ-potential,

(1) ULJ(r)=4εαβσαβr12−σαβr6

where α,β=A,B. The system is composed by fractions of (80:20) of A- and B-type particles. Interaction parameters are given by $\varepsilon_{AA}=1$, $\varepsilon_{AB}=1.5$, $\varepsilon_{BB}=0.5$, $\sigma_{AA}=1$, $\sigma_{AB}=0.8$, $\sigma_{BB}=0.88$ and $m_{A}=m_{B}=1$. In the following, all units are given in reduced form, i.e., energies, lengths, and masses are given in units of $\varepsilon_{AA}$, $\sigma_{AA}$ and $m_{A}$. Accordingly, times are given in units tLJ=σAAmAεAA and temperatures in TLJ=εAAkB with the Boltzmann constant $k_{B}$. The interaction potential is truncated at a distance $r_{c}=2.245$ being twice the minimum range of $U_{\mathrm{LJ}}$. We integrate equations of motion for this model system using LAMMPS (Large-scale Atomic/Molecular Massively Parallel Simulator) [28] using a time step of dt=0.005.

The above described model has shown its reliability in terms of reproducing many phenomena particularly important to the generic behavior of glasses such as the glass transition in both the quiescent state [27,29,30,31,32], under mechanical load [25,33,34,35], and in describing the system Ni${}_{80}$Pd${}_{20}$—a metal–metaloid glass—correctly on a qualitative level [36,37]. It is also noteworthy that, recently, the phase diagram of this binary LJ mixture for the entire range of B-particle concentrations has been worked out [38] and is related to local structure [39].

Two types of simulation boxes—one slab-shaped and a cubic box—are considered in this study. The slab-shaped box has linear dimensions of $L_{x}\times L_{y}\times L_{z}=2000\times 10\times 100$ and contains $N_{\mathrm{slab}}=2.4\times 10^{6}$ particles. The cubic box, on the other hand, has a side length of $L_{x}=L_{y}=L_{z}=127.72$ with $N_{\mathrm{cube}}=2.5\times 10^{6}$. Thus, the number density in both cases is ρ=1.2. As this dense packing, the (80:20)-Kob–Andersen model shows a glass transition at $T_{g}\approx 0.41$ [34,40]. Throughout all simulations in this study, particle number and volume are kept constant and the temperature is controlled via a Nosé–Hoover thermostat (NVT-ensemble).

As to the preparation protocol, the system is first brought to thermal equilibrium in the liquid state at T=1. At this temperature, the mean square displacements show that the center of an A-type particle explores a spatial domain comparable to its own size within a time of roughly $t_{\mathrm{MSD}=1}\approx 100t_{\mathrm{LJ}}$ [27]. Taking this as a typical relaxation time, we equilibrate the system at T=1 for a time of $1000\;t_{\mathrm{LJ}}$ and then store snapshot every $600\;t_{\mathrm{LJ}}$ on disc. This way we generate four statistically independent configurations. These samples are quenched to a temperature $T=0.2\approx 0.49\;T_{g}$ with a rate $\dot{T}=10^{-2}$. In order to produce well-aged samples with relaxation times well beyond the duration of a thermal cycle, we let the thus obtained configurations undergo aging at a constant temperature of T=0.2 for $t_{\mathrm{age}}=5\times 10^{4}t_{\mathrm{LJ}}$.

### 2.2. Thermal Treatment

After the above described preparation, a periodic temperature variation is imposed on the samples between the initial value of $T_{i}=0.2\approx 0.49\;T_{g}$ and the cryogenic value of $T_{0}=10^{-4}\approx 0.00024\;T_{g}$. The cycling period, i.e., the duration of one thermal cycle, is chosen to be $\tau_{\mathrm{tc}}=500$. This time is sufficiently long for local thermal expansion and contraction to become effective and at the same time short enough so that aging processes do not play a major role during one single cycle (recall that the waiting time prior to thermal treatment, $t_{\mathrm{age}}=5\times 10^{4}$, is by a factor of 100 larger than the time for a single cycle).

**Figure 1:**
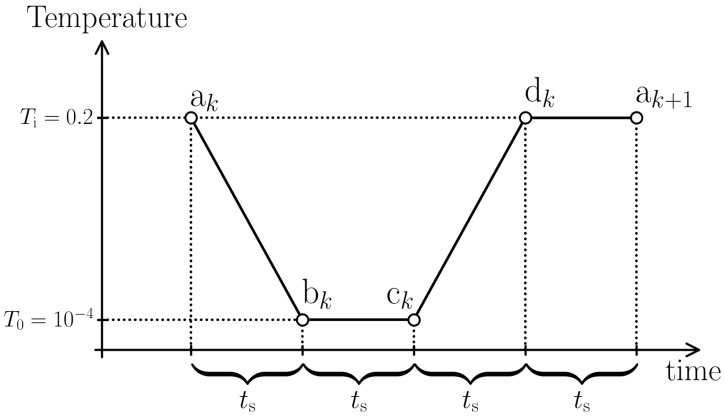
Schematic view of the imposed cyclic temperature variation. The letter $a_{k}$ denotes the initial state of the *k*th cycle and $b_{k}$ refers to the state right after cooling. $c_{k}$ marks the time after holding the temperature constant at $T=T_{0}=10^{-4}$ and $d_{k}$ is the first instant after reheating. The cycle ends at $a_{k+1}$ after a holding step at $T=T_{i}=0.2$. Within this notation, the initial “as-aged” state, which we specify with the index *i*, coincides with $a_{1}$.

In our protocol, each thermal cycle consists of four consecutive steps, each with a duration of $125\;t_{\mathrm{LJ}}$. For the *k*th, cycle these four steps are: (i) cooling from $T_{i}=0.2$ to $T_{0}=10^{-4}$ ($a_{k}\to b_{k}$ in Figure 1), (ii) holding the temperature constant at $T_{0}$ ($b_{k}\to c_{k}$), (iii) heating from $T_{0}$ to $T_{i}$ ($c_{k}\to d_{k}$), and (iv) holding the temperature constant at $T_{i}$ ($d_{k}\to a_{k+1}$). Heating and cooling rates during thermal cycling are thus given by $\dot{T}=\pm 1.6\times 10^{-3}\;t_{\mathrm{LJ}}$. The total number of cycles is n=50.

For further reference, the initial “as-aged” configuration prior to thermal cycling, i.e., the one obtained after the long aging process is denoted with index *i*. For a more detailed scrutiny, we select four distinct points along a thermal cycle and denote them as follows. The starting point of each cycle is marked by $a_{k}$, where *k* counts the number of cycles. The state after cooling is referred to by $b_{k}$, after the subsequent holding at the same (low) temperature by $c_{k}$ and after heating by $d_{k}$. Obviously, the *k*th cycle ends at $a_{k+1}$.

## 3. Results

**Figure 2:**
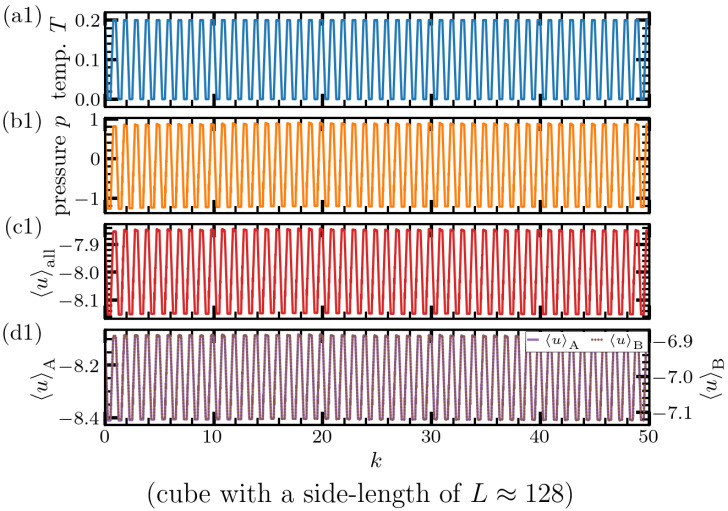

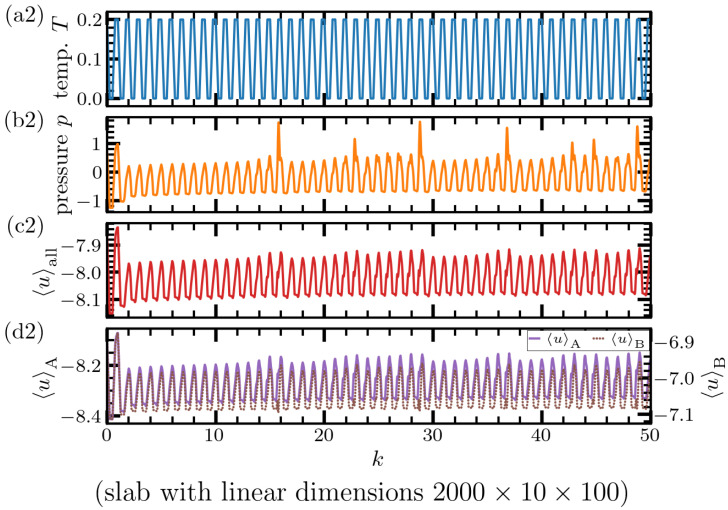
Temperature (**a**), pressure (**b**), and per-particle potential energy of all (**c**) and respective atoms types (**d**) during 50 thermal cycles with a cycling period $\tau_{\mathrm{tc}}=500$. The upper image (**a1**–**d1**) shows the result for a cubic simulation box with a linear dimension of roughly 128 particle diameters. The lower image (**a2**–**d2**) corresponds to the slab geometry, where the largest linear dimension reaches 2000 particle diameters. In the case of cube *T*, *p* and 〈u〉 follow the prescribed cyclic variation of temperature, which is imposed by the Nosé–Hoover thermostat. In marked contrast to this, the slab develops a significantly different response. Both pressure and per-particle energy develop a more complex time dependence, with small-amplitude oscillations at the frequency of the imposed cyclic perturbation modulated by slower amplitude-variations. In the case of pressure (**b2**), irregular jumps are observed, indicative of random release of the energy, which is accumulated over the course of preceding cycles. The fact that kinetic energy follows the prescribed curve is reassuring and shows that thermal cycling is sufficiently slow for the relaxation of kinetic energy to the prescribed value.

To gain a first idea about the effects of thermal cycling, we show in Figure 2 instantaneous temperature, pressure, and energy per-particle for all and A-type particles. Here, instantaneous temperature is calculated via $T=\frac{m}{3Nk_{B}}\sum_{i=1}^{N}v_{i}^{2}$, where $k_{B}$ is the Boltzmann constant, *N* the total number of particles, and $v_{i}$ is the velocity of *i*th particle. Pressure is obtained via the standard virial theorem, which yields $p=\frac{Nk_{B}T}{V}+\frac{1}{3V}\sum_{i=1}^{N}r_{i}\cdot f_{i}$, where *V* is the system volume, $r_{i}$ the position of particle *i*, and $f_{i}$ the total force acting on it. The mean potential energy per-particle is calculated via ${\langle u\rangle }_{\alpha }=\frac{1}{N_{\alpha }}\sum_{i=1}^{N_{\alpha }}u_{i}$, where $u_{i}$ is the interaction potential energy of particle *i* with all its neighbors within the cutoff distance. The per-particle energy is calculated for all particles irrespective of their type (α=‘all’), and separately for A-type (α=‘A’) and B-type (α=‘B’).

As illustrated in Figure 2, for the case of a cube with a linear dimension of roughly 128 particle diameters, instantaneous temperature, pressure, and energy per particle follows the prescribed cyclic perturbation imposed by the Nosé–Hoover thermostat. The response, which develops inside the cubic simulation box, is regular, and it is difficult to see from this plot a significant effect of thermal cycling. Inline with our previous report [20], this first estimate on the lack of a strong effect in the cubic box is corroborated by a more detailed analysis below, where the variance is found to increase slightly while at the same time average energy per particle decreases.

In contrast to this weak sensitivity of the cubic box, a cyclic temperature variation leads to a considerably different response in the case of a slab-shaped system with roughly 15 times larger linear dimension. In this case, both pressure and per-particle energy exhibit a time dependence, where small-amplitude oscillations at the imposed thermal cycling frequency are superimposed with slower variations of amplitude. It is also interesting to mention the rather irregular pressure jumps observable in Figure 2b2. These irregularities are indicative of the sudden release of energy that has been accumulated over the course of the preceding cycles. This interpretation is inline with the fact, after such jumps in pressure, that the average potential energy (Figure 2c2) shows a slight decrease. A reason for this irregular response could be that cyclic expansion and contraction couples effectively to structural heterogeneity in the system. It is thus interesting to examine whether and to which extent this perturbation is capable of “rejuvenating” the system.

After stopping thermal cycling, pressure (and potential energy) oscillations can be observed. To not introduce bias into our analysis, for this purpose, after stopping the thermal cycling process, we let the system evolve in time at constant temperature and monitor pressure and energy until they become roughly time-independent.

**Figure 3:**
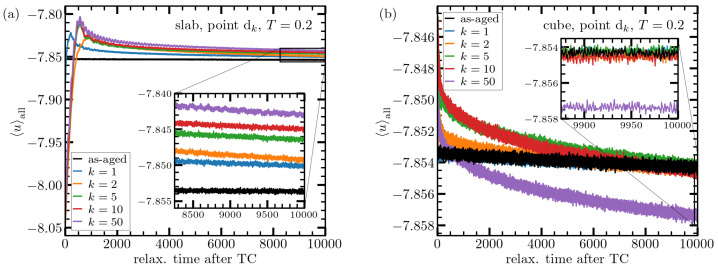
Time evolution of per-particle potential energy for the slab (**a**) and cube (**b**) geometry after stopping thermal cycling at point $d_{k}$ for various *k* as indicated. The black line gives the data for an “as-aged” system, i.e., a configuration that was not at all subject to thermal cycling but just evolved with time in parallel to the cycled samples. Just after stopping temperature change (t=0), energies are well below this black line. The reason is that, during thermal cycling, the system was regularly exposed to temperatures between $T_{i}=0.2$ and $T_{0}=10^{-4}$, with an average of $\approx T_{i}/2$. It thus needs time to relax toward states that correspond to the higher temperature, $T_{i}$. Interestingly, this relaxation process establishes on long times an energy higher than that of the unperturbed sample. This raise in energy is enhanced within the first cycles but then saturates after roughly five cycles. The inset shows a close-up of the same data on long times. Note the amplitude of energy variations is roughly twenty times larger in the slab as compared to the cube. The seemingly large decrease of energy for the cube at k=50 is probably due to fluctuations in heterogeneity. Indeed, the relative magnitude of the difference in energy between k=50 and other cycles is roughly 0.05%, which is quite close to the relative size of statistical uncertainty, $1/\sqrt{N_{\mathrm{cube}}}=1/\sqrt{2.5\times 10^{6}}\approx 0.06\%$.

The left panel in Figure 3 depicts for the case of the slab the temporal evolution of energy per particle at constant temperature after stopping thermal cycling at point $d_{k}$ (i.e., after *k* cycles). Since the system has been subject to temperatures well below $T_{i}$ during thermal cycling, its energy has effectively decreased in the course of thermal perturbation. This fact explains why energy of the cycled samples all start below the black line, which corresponds to a system which instead of being thermally cycled, has just undergone the aging process. This time evolution process already reveals a first signature of rejuvenation as the system reaches energies that are higher than that of the unperturbed sample. The largest contribution to this rejuvenation effect arises from the first cycle and is enhanced further by the next few cycles, saturating finally at roughly five cycles. It is also seen that the time evolution of energy slows down significantly and approaches a quasi-plateau. This allows us to use these late configurations and determine their energy distributions.

This strong effect on the energy of the long system (slab) must be contrasted to the weak perturbation caused by deep thermal cycling in the shorter system (cube). As seen from the right panel of Figure 3, the change in the energy of the cubic system is roughly 20 times smaller than that of the slab. This is inline with our previous report on the insensitivity of the cubic system to thermal perturbations. Since the number of particles between the two cases differs only by roughly 4%, the origin of this increase in sensitivity to a cyclic temperature variation does not lie in the particle number but presumably it is the linear system dimension that plays a key role here.

**Figure 4:**
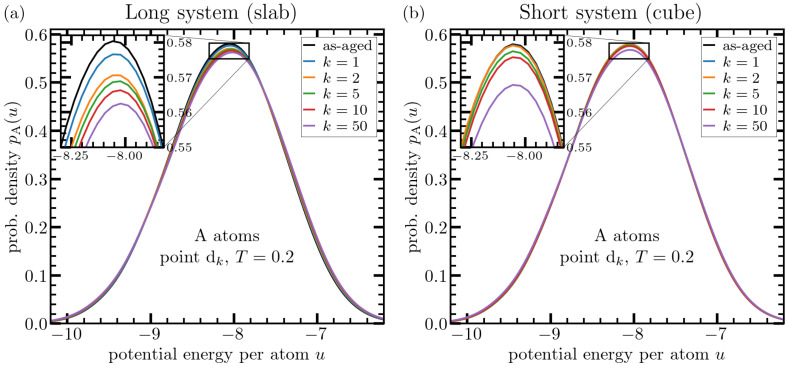
Effect of deep thermal cycling on probability distribution for energy per particle for A-type, evaluated at point $d_{k}$. Panel (**a**) shows the data for a system with a linear dimension of $L_{\mathrm{slab}}=2000$ particle diameters (slab geometry) and panel (**b**) depicts the same type of data for a cubic system with $L_{\mathrm{cube}}=128$. Different curves correspond to different number of thermal cycles as indicated in the legends. Prior to data analysis, thermal cycling is stopped at point $d_{k}$ and the system is relaxed for a duration of $10^{4}t_{\mathrm{LJ}}$ at $T=T_{i}=0.2$. This serves to damp pressure waves, which are produced due to thermally-induced expansion and contraction. Even though somewhat difficult on this scale, a broadening of energy distribution can be seen in panel (**a**) (see also Figure 7) indicating increased heterogeneity due to thermal cycling, i.e., rejuvenation. The inset serves to highlight the shift in mean energy toward higher values upon thermal cycling. This shift is much smaller in the right panel (cube).

As mentioned above, we determine per-particle energy distributions after damping the sound waves. We apply the same preparation and analysis protocol both to the slab and cubic systems. This means that the same damping time for pressure waves is applied in the both cases prior to energy sampling. The obtained histograms of A-type particles are depicted in Figure 4 for both systems, slab and cube. While a broadening of the energy distribution can be discerned (albeit after some careful scrutiny) in the case of slab, the cube-data rather hint towards a weak effect on the width of the distribution function. As to the mean energy, in the slab, it is clearly shifted to larger values upon deep thermal cycling. Both these features hint toward an enhanced heterogeneity in structure. In contrast to the slab, an evaluation of the first moment of energy distribution reveals that the mean energy decreases upon thermal cycling for the cubic system, whose linear dimension is roughly fifteen times smaller than the slab-length.

**Figure 5:**
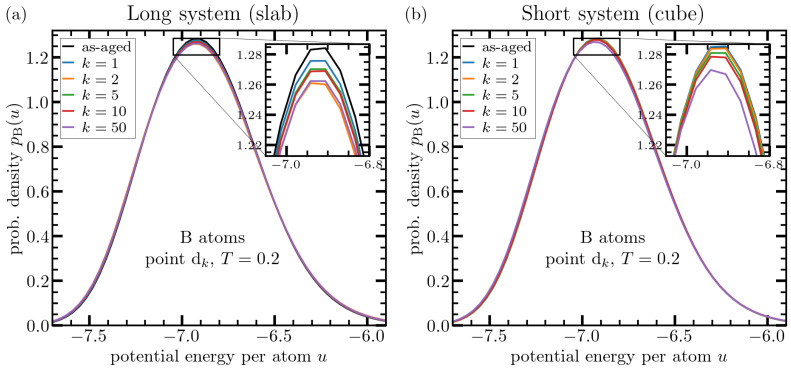
The same data as in Figure 4 for B-type particles. Panel (**a**) shows data for a system with a linear dimension of $L_{\mathrm{slab}}=2000$ particle diameters (slab geometry), panel (**b**) depicts similar data for a cubic system with $L_{\mathrm{cube}}=128$. Different curves correspond to different number of thermal cycles as indicated in the legends. In the case of B-particles, it is still more difficult to make a judgment based on the overall shape of the distribution.

**Figure 6:**
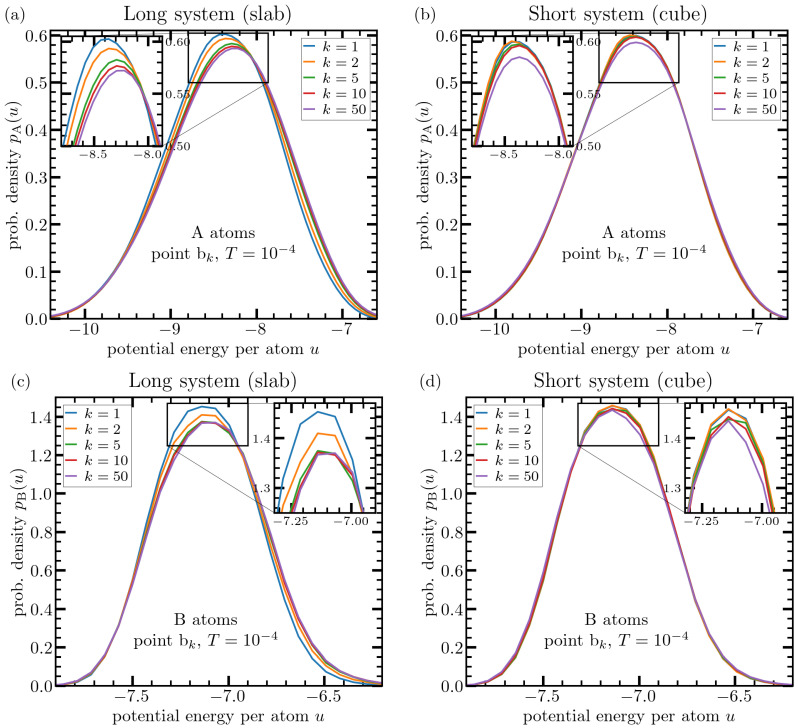
Similar type of analysis as in Figure 4 evaluated at point $b_{k}$, i.e., at the cryogenic temperature, where the system structure is essentially frozen due to the lack of kinetic energy. Upper panels show $p_{\alpha }\left(u\right)$ as obtained after applying different numbers of thermal cycles as indicated in the legends for A-atoms in the case of of the slab (**a**) and cube (**b**) geometry. Panels (**c**,**d**) show similar data for the case of B-atoms. In this case, the crucial difference between long and short systems regarding the effects of deep thermal cycling is more apparent.

**Figure 7:**
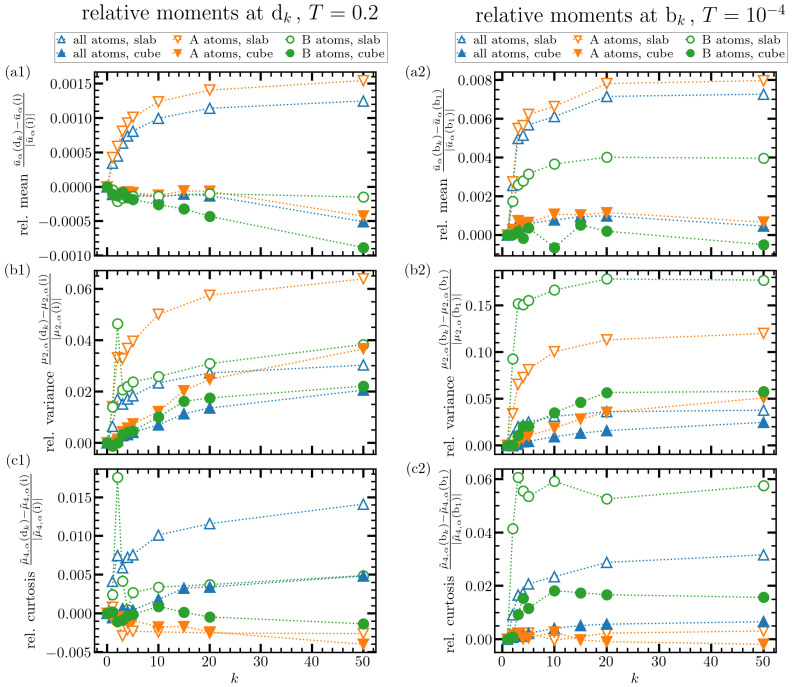
Moments of per-atom potential energy distributions. Relative mean variance and curtosis of the per-particle potential energy distributions after stopping TC at points $d_{k}$ (**a1**–**c1**), $b_{k}$ (**a2**–**c2**), respectively. An additional relaxation time of $10^{4}\;t_{\mathrm{LJ}}$ has been applied before evaluating potential energies to account for the damping of TC induced pressure oscillations. Data for A-particles, B-particles and all particles can be found as indicated in the legends. In all the cases shown, the strongest TC-effects occur for the lower cycling temperature ($T=10^{-4}\approx 0.00024T_{g}$) and in the longer system (slab).

As to the effects of particle type, we generally observe a stronger effect of cryogenic thermal cycling on A-type quantities as compared to those related to B-particles (see, e.g., Figure 5, Figure 6 and Figure 7). This behavior may be related to the fact that A-particles are both larger and four times more in number and thus build the structural backbone of the system. The fewer and smaller B-particles are more flexible and can adapt themselves to the changes of the overall structure. They can thus reach a lower energy state more easily than the larger and less mobile A particles. Nevertheless, a broadening due to a more heterogeneous structure can be observed for B-atoms, too (Figure 7b1,b2).

We have repeated the same type of analysis whose results are shown in Figure 4 and Figure 5, for a starting point in the cryogenic-temperature domain. For this purpose, we have stopped thermal cycling at the point $b_{k}$ and have waited again a fairly long time of $10^{4}t_{\mathrm{LJ}}$ to allow for damping of sound waves. Results on per-particle energy distribution obtained from these new state points are compiled in Figure 6.

These data underline the above discussed difference in response to deep thermal cycling in the case of long and short systems (slab versus cube) in a more striking manner. Now, B-particles are more sensitive to the thermal treatment. This can be rationalized by the fact that a temperature of $T=10^{-4}$ is essentially equivalent to complete kinetic arrest of all particles (recall that $T_{g}=0.41$ for the present system). Thus, not only A-particles are immobilized but also the mobility of B-particles is almost completely suppressed so that they can no longer explore the free spaces as they could at a significantly higher temperature of $T_{i}=0.49T_{g}$.

Noteworthy, in all the cases investigated, the strongest rejuvenation effect arises from the first few cycles and saturates rather rapidly upon further cycling. This fact is made quantitative in Figure 7, where various moments, both absolute and relative to the mean value, are evaluated and depicted versus cycle number. The quantities shown in this figure are evaluated as follows. The central quantity here is the per-particle energy distribution, $p_{\alpha }\left(u\right)$, where α∈{A,B} is the particle type. This function is normalized such that $\int p_{\alpha }\left(u\right)\;du=1$. We then define the mean energy as the first moment of the per-particle distribution function

$${\bar{u}}_{\alpha }=\int p_{\alpha }\left(u\right)udu\;.$$

Using this result, we further define the variance $\mu_{2,\alpha }$ and standard deviation ${\mathrm{sd}}_{\alpha }$ via

$$\mu_{2,\alpha }=\int {\left(u-\bar{u}\right)}^{2}p_{\alpha }\left(u\right)du\;\;\;\mathrm{and}\;\;\;{\mathrm{sd}}_{\alpha }=\sqrt{\mu_{2,\alpha }}\;.$$

In order to access tails of the distribution function, we also evaluate the fourth moment and curtosis,

$$\mu_{4,\alpha }=\int {\left(u-\bar{u}\right)}^{4}p_{\alpha }\left(u\right)du\;\;\;\;\mathrm{and}\;\;\;\;{\tilde{\mu }}_{4,\alpha }=\frac{\mu_{4,\alpha }}{{\mathrm{sd}}_{\alpha }^{4}}\;.$$

Since we are interested in changes of these quantities upon temperature treatment, we define their relative variation compared to a reference state (Figure 1):

(2) $$\mathrm{relative}\;\mathrm{mean}\;=\frac{{\bar{u}}_{\alpha }\left(d_{k}\right)-{\bar{u}}_{\alpha }\left(i\right)}{|{\bar{u}}_{\alpha }\left(i\right)|},$$

(3) $$\mathrm{relative}\;\mathrm{variance}\;=\frac{\mu_{2,\alpha }\left(d_{k}\right)-\mu_{2,\alpha }\left(i\right)}{|\mu_{2,\alpha }\left(i\right)|},$$

(4) $$\mathrm{relative}\;\mathrm{curtosis}\;=\frac{{\tilde{\mu }}_{4,\alpha }\left(d_{k}\right)-{\tilde{\mu }}_{4,\alpha }\left(i\right)}{|{\tilde{\mu }}_{4,\alpha }\left(i\right)|}.$$

Obviously, the initial point *i* cannot be used as a reference when evaluating the effects of thermal cycling at the cryogenic temperature $T=10^{-4}\approx 0.00024T_{g}$. In this case, the state $b_{k}$ is compared to $b_{1}$.

Figure 7 depicts results on the above defined quantities, Equations (2)–(4), for the data evaluated at the upper (left panels in Figure 7) and lower (right ones) cycling temperatures of $T_{i}=0.2\approx 0.49T_{g}$ and $T_{0}=10^{-4}\approx 0.00024T_{g}$. At T=0.2, the mean energy decreases upon thermal treatment in the cubic system. In the slab, A-particles are clearly exited to higher energies, but B-particles—being smaller and having a preference for the proximity of A-particles—seem to be mobile enough to explore the available (interstitial) free volume deeper and find energy states. A-particles being the majority component, the per-particle-energy of the entire system also shows signature of this excitation. Thus, judging by the mean energy alone, one could say that thermal treatment rejuvenates the system with a longer linear dimension (the slab), but the shorter simulation box shows relaxation. A scrutiny of the mean energies at the lower cycling temperature ($T=10^{-4}$) in Figure 7a (right panel) reveals, however, that a certain, rejuvenation, albeit weaker than in the slab is present also in the cubic system. Presumably, this effect is not sufficiently strong to compete with the relaxation processes at the higher temperature of $T=0.49T_{g}$. In the case of variance of the energy distribution, however, this competition seems to be more in favor of rejuvenation tan relaxation: Variance is apparently enhanced (signaling a broader per-particle energy distribution) in all the investigated cases. This effect is quite strong at $T=10^{-4}$ (Figure 7b, right panel), becomes weaker at T=0.2 (Figure 7b, left panel) due to a faster relaxation but is still strong enough to be visible both in the slab and in the cube.

A comment is at order here. In our previous study [20], this relatively weak rejuvenation effect in the cubic system was suppressed by two additional factors. First, the cube had twice less particles as in the present study, thus leading to less strong effects of heterogeneity. Second, the cycling frequency was ten times lower so that relaxation had ten times more time to oppose rejuvenation. Therefore, in that study, a weak decrease of variance was observed (see Figure 12a in [20]). From the present set of data, however, it is clearly seen that it is possible to tune the thermal cycling parameters such that a detectable rejuvenation occurs.

The broadening of energy distribution upon thermal cycling also manifests itself in curtosis, which is apparently enhanced upon thermal treatment, Figure 7c. It is noteworthy that curtosis of a bimodal distribution function (the one corresponding to ‘all atoms’ in Figure 7c) is rather difficult to interpret. A survey of A-particle and B-particle curtosis, however, clearly reveals an increase due to thermal treatment. Again, and in agreement with the behavior of mean energy and variance, a comparison of the left and right panels in Figure 7c reveals that, the rise in curtosis is more enhanced at the lower cycling temperature of $T=10^{-4}$.

## 4. Conclusions and Outlook

In this study, we have investigated the effect of low temperature (cryogenic) thermal cycling on per-particle energy distribution of a standard model glass, the well-known 80:20 binary Lennard-Jones mixture, first introduced by Kob and Andersen in their seminal work [26,27].

The background of this study is that we had addressed in a previous publication [20] the possibility of rejuvenation of the glassy state upon a cyclic temperature variation well below the glass transition point but had only found that rejuvenation processes were not strong enough to stop or reverse aging but could only slow it down. In the same manuscript, this observation was corroborated on a qualitative level by calorimetry experiments on bulk metallic glasses. Diffusion measurements in a pre-deformed bulk metallic glass, however, show that cryogenic thermal cycling may accelerate diffusion, at least if the sample has undergone a plastic deformation prior to thermal treatment [24].

This work represents a second attempt to unravel rejuvenation in our model glass induced by deep thermal cycling. For this purpose, we have tuned two important parameters: On the one hand, we have increased the aging time prior to cycling (thus producing a more relaxed initial state) and at the same time enhanced the cycling frequency. Thereby, we enlarged the separation of time scales between aging and thermal cycling. On the other hand, we have increased the linear dimension of the simulation box by roughly a factor of fifteen to better explore effects arising from structural heterogeneity. The increase in system length was not achieved by a brute force increase of the particle number in a cube geometry—which would have been computationally prohibitively expensive—but by using a slab geometry, with which we had already good experience in dealing with structural heterogeneity in shear bands [25].

When combined with our previous report [20], the present study shows that, depending on size, linear dimension and the rate of cycling, rejuvenation can be clearly observed in per-particle energy distribution. This is manifest both in a shift of the mean per-particle energy to higher values and in a broadening of the shape of the distribution function. Simulations clearly show that rejuvenation effects are much stronger in the system with a larger linear dimension (slab) than in the shorter one (cube). Noting that the number of particles in the both systems is roughly equal ($N_{\mathrm{cube}}=2.5\times 10^{6}$ versus $N_{\mathrm{slab}}=2.4\times 10^{6}$), our observation emphasizes the importance of linear system dimension for the development of strong stresses upon thermal cycling as compared to purely volumetric effects.

The present work is just a starting point to explore effects of cryogenic thermal cycling on properties of bulk metallic glasses. An important issue to be directly addressed in future computer simulations and experiments regards mechanical properties. After all, one of the main motivations for introducing deep thermal treatment of metallic glasses was the idea that it could lead to an improvement of ductility in this important class of materials [13]. A simple possibility here, at least in computer simulations, would be to investigate the response to a shear deformation prior to and after a thermal treatment. Another interesting issue, motivated by recent experiments [24], is effects of deep thermal cycling on tracer diffusion. Indeed, both experimentally and from the molecular dynamics side, little is known about how diffusion is influenced by cryogenic thermal cycling. The main obstacle in simulation studies of diffusion is, of course, the slow dynamics in the glassy state. In this regard, it would be highly desirable to develop new algorithms to access not only stable glass states [41] but also to follow the dynamic trajectory for long times.

## Data Availability

Data sharing is not applicable to this article.
